# Yeast screening and cell immobilization on inert supports for ethanol production from cheese whey permeate with high lactose loads

**DOI:** 10.1371/journal.pone.0210002

**Published:** 2018-12-31

**Authors:** Rebeca Díez-Antolínez, María Hijosa-Valsero, Ana I. Paniagua-García, Jerson Garita-Cambronero, Xiomar Gómez

**Affiliations:** 1 Center of Biofuels and Bioproducts, Instituto Tecnológico Agrario de Castilla y León (ITACyL), Villarejo de Órbigo, León, Spain; 2 Chemical and Environmental Bioprocess Engineering Group, Natural Resources Institute (IRENA), University of León, León, Spain; CNR, ITALY

## Abstract

Eight yeast strains of the genera *Saccharomyces* and *Kluyveromyces* were screened to ferment high lactose-load cheese whey permeate (CWP) (>130 g/L lactose) without nutrient supplementation. The fermentation conditions (temperature, pH and time) were optimized to maximize the fermentation performance (ethanol titer, ethanol yield and lactose consumption) for the two preselected strains, *K*. *marxianus* DSM 5422 and *S*. *cerevisiae* Ethanol Red, using a response surface methodology (RSM). Under optimized conditions, *K*. *marxianus* DSM 5422 attained ethanol titers of 6% (v/v) in only 44 h. Moreover, the feasibility of immobilizing this strain on four different inorganic supports (plastic, glass and Tygon silicone Raschig rings and alumina beads) was assessed. Glass Raschig rings and alumina beads showed a more stable performance over time, yielding ethanol titers of 60 g/L during 1,000 hours, which remarkably reduces yeast cultivation costs. Results demonstrate the feasibility of using CWP for successful ethanol production in a simple and economical process, which represents an attractive alternative for waste treatment in dairy industries.

## Introduction

Cheese whey, the liquid by-product of milk coagulation during cheese production, is the most important source of organic contamination in the dairy industry due to the large volumes produced. About 10 L of cheese whey are generated for each kilogram of cheese manufactured [1, 2]. It contains about 50% of the total solid content of the original milk, with lactose (48–60 g/L), proteins (6–8 g/L) and mineral salts (4–10 g/L) as major components [3]. The European Union (EU-28) and USA are the largest producers of cheese, generating more than 155 Mton each year [4]. Cheese whey is characterized by a high organic pollutant load with high biological and chemical oxygen demand values (BOD and COD) ranging between 40–60 g/L and 50–80 g/L, respectively [5]. Lactose is responsible for 90% of the COD and BOD contents in whey [3]. About 50% of the cheese whey production is treated or valorized as source of proteins and lactose into feed and food products [6]. However, the surplus of lactose is not further resourceful; consequently, whey disposal means a serious environmental and economic problem [7].

The improper disposal of whey may cause major environmental problems like eutrophication or toxicity in the receiving environments [1]. Therefore, environmental restrictive rules have been established, forcing the dairy industry to find solutions to the large whey volumes generated and to seek for alternatives rather than the direct discharge. Nowadays, whey is evolving into a sought-after product because of the nutrients it contains and the functional properties it imparts to food [8]. Moreover, its use as substrate for the biological production of several value-added products such as single cell protein, solvents (e.g. ethanol, butanol or acetone), organic acids (e.g. acetic, butyric, lactic, malic, propionic, malic or succinic), hydrogen, biopolymers and biodegradable plastics [1, 7] has been proposed.

Cheese whey has been employed as low cost and abundant raw material substrate for ethanol production. However, the alcoholic fermentation of whey is hardly economically competitive in comparison to traditional feedstocks such as sugar cane or corn [9]. Despite this fact, the biotechnological reuse of this abundant and widely spread waste as a source for fuel production offering no competition with the food market and land uses is strongly desirable [10].

Not many yeast strains are capable of naturally fermenting lactose to ethanol. Traditional yeasts used for industrial fermentation processes, such as *Saccharomyces cerevisiae*, cannot metabolize lactose, due to the lack of both lactose permease and β-galactosidase enzyme systems [11]. Therefore, an enzymatic or chemical hydrolysis of lactose is required to use whey for ethanol production using *S*. *cerevisiae*. Typical ethanol yields from lactose are reported as 80–85% of theoretical [12] when using cheese whey with lactose concentrations of 40–50 g/L. Another alternative strategy is the engineering of *S*. *cerevisiae*, but most of the obtained strains have shown undesirable characteristics such as low growth, genetic instability and low ethanol production [9, 13].

Nevertheless, most of the *Kluyveromyces* species are capable of metabolizing lactose to ethanol. In spite of the interesting attributes of *Kluyveromyces* species, such as thermotolerance, high growth rate, capacity to metabolize a wide variety of carbohydrates such as hexoses, pentoses and disaccharides [14] and its GRAS (generally recognized as safe) status for protein production [15, 16], it is far away to compete in industrial processes using *Saccharomyces*.

Different strategies have been tested for developing ethanol production processes using these species. For *K*. *marxianus*, Gabardo *et al*. [17] concluded that it is unnecessary to supplement either whey or whey permeate because this organic stream is already rich in nutrients and the addition of nitrogen could affect ethanol production by cell metabolism impairment. Nevertheless, inhibitory problems or process imbalances have been frequently reported when working with substrate concentrations higher than 100 g/L [17–20]. Therefore, direct fermentation of whey is not economically feasible due to low ethanol concentrations and high distillation costs [19, 20]. Nevertheless, Díez-Antolínez *et al*. [21] reported the viability of directly fermenting non-supplemented cheese whey permeate (CWP) with a lactose load of about 170 g/L without substrate inhibitions, reaching ethanol yield efficiencies of 95.5% in 48 h with ethanol titers of 86.6 g/L.

In the case of *S*. *cerevisiae*, the conversion of galactose into glycolytic intermediates needs energy and additional catabolic steps, because glycolytic enzymes are not galactose-specific. Hence, the Leloir pathway is switched on to convert galactose into glucose 6-phosphate, metabolized in the glycolysis pathway and reduced to ethanol [13, 22, 23]. Therefore, *S*. *cerevisiae* strains require richer nutrient media, especially in nitrogen, to efficiently convert galactose into ethanol, reporting ethanol concentrations of only 18 g/L and 15 g/L using as substrates non-supplemented cheese whey and cheese whey permeates, respectively [13].

Due to the low productivity of batch ethanol fermentation, continuous processes based on the immobilization of *S*. *cerevisiae* and *K*. *marxianus* to increase cell concentration in organic and inorganic supports [21, 24, 25] have been studied. Cell immobilization techniques overcome most of the bioprocess restrictions. They offer long-term cellular stability, increased molecular selectivity, higher resistance against inhibition, better cell protection against the environment, more active biocatalyst surface per unit of reactor volume, low loss of activity during immobilization and fermentation, reduced lag phase and shorter reaction time; as a result, the reactor design is more efficient [26–28]. Moreover, cells can be used repeatedly and continuously, which helps to maintain a high cell density during the whole process, thus reducing the cost of the bioprocess [26]. However, cell immobilization techniques present disadvantages such as reduction on accessibility to the substrate, alterations in biocatalyst conformation and activity, stress problems on biocatalysts, or more costly specific reactor systems with high-engineering design [26]. Among immobilization applicable methods, entrapment or encapsulation of cells within a gel or adsorption on a solid support are the most common and effective ones [29]. Gel supports have great problems of stability over time [30–31] and organic supports require complex derivatization pretreatments [32]. Thus, it is interesting to explore the viability of inorganic supports in alcoholic fermentations.

The main aim of this work was to compare the ability of eight yeast strains of the genera *Saccharomyces* and *Kluyveromyces* to ferment high lactose-load cheese whey permeate (CWP) and to select the most efficient strain for the production of ethanol and optimize operating conditions (temperature, initial pH and time) to maximize the fermentation performance. To the best of our knowledge, no study has been reported regarding the use of *K*. *marxianus* strains immobilized on inert supports for alcoholic fermentations tested during 1,000 hours by repeated-batch recycling.

## Material and methods

### Raw material

The CWP used as substrate was obtained from a mixture of cow and sheep milk after a concentration process by ultrafiltration and it was provided by Quesería Entrepinares SAU (Valladolid, Spain). CWP was pasteurized by heating at 80°C for 30 min to eliminate endogenous microorganisms. Lactose content ranged between 120–170 g/L with a protein content of 34 g/L. The CWP presented an initial pH of 5.8.

### Yeast strains and culture conditions

Four strains of *K*. *marxianus* and four strains of *S*. *cerevisiae* were used in this work. *K*. *marxianus* DSM 5418, DSM 5422, DSM 7239 and DSM 70799 were provided in lyophilized form by Deutsche Sammlung von Mikroorganismen und Zellkulturen GmbH (Braunschweig, Germany). The reactivated culture was maintained on nutrient agar and stored at 4°C. A loopful of a slant culture was transferred to sterilized growth medium [50 g/L lactose (Sigma Aldrich, Steinheim, Germany), 0.3 g/L MgSO_4_·7H_2_O (Sigma Aldrich), 5 g/L yeast extract (AES Laboratories, Bruz, France), 2 g/L NH_4_Cl (Fluka-Sigma Aldrich, Steinheim, Germany), 10 g/L peptone (Fluka, Buchs, Switzerland) and 1 g/L KH_2_PO_4_ (Panreac, Castellar del Vallès, Spain)]. The medium was incubated in an orbital shaker Infors HT Minitron (Bottmingen, Switzerland) at 35°C with a constant shaking at 120 rpm during 7 h in order to obtain exponential-phase cells.

Four *S*. *cerevisiae* strains with industrial applications were chosen. The freeze-dried distillery yeast *S*. *cerevisiae* Ethanol Red (Lesaffre Company, Marcq-en-Baroeul, France), the new osmotolerant hybrid *S*. *cerevisiae* CECT 13152 (kindly provided by Tomsa Destil S.L., Madrid, Spain) obtained from the protoplast fusion of *S*. *cerevisiae* NCYC73 and a non-identified strain of *S*. *cerevisiae*, the compressed baker’s yeast branded *S*. *cerevisiae* Hércules (Lessafre Ibérica S.A., Valladolid, Spain) and the distillery yeast *S*. *cerevisiae* CECT 1383 provided by Colección Española de Cultivos Tipo (Valencia, Spain) were used in this study. In the case of strains Ethanol Red, CECT 13152 and Hércules, 0.1% (w/v) yeast was directly added to the fermenter without a previous propagation step. Strain CECT 1383 was reactivated, maintained on nutrient agar and stored at 4°C. A loopful of a slant culture was transferred to sterilized growth medium [20 g/L glucose (Sigma Aldrich, Steinheim, Germany), 10 g/L of yeast extract (AES Laboratories, Bruz, France) and 10 g/L peptone (Fluka, Buchs, Switzerland)]. The medium was incubated in an orbital shaker Infors HT Minitron (Bottmingen, Switzerland) at 32°C with a constant shaking at 120 rpm during 7 h in order to obtain exponential-phase cells. A viable cell concentration of about 10^8^ cells/mL was obtained.

### *Kluyveromyces* strains and fermentation media comparison

In order to find the most suitable yeast strain for directly fermenting high-loaded CWP to ethanol, the four *K*. *marxianus* strains listed in section *Yeast strains and culture conditions* were assessed employing a synthetic medium with an initial lactose concentration of 130 g/L, supplemented with 3 g/L yeast extract, 2 g/L peptone, 2 g/L NH_4_Cl, 2 g/L KH_2_PO_4_, 2 g/L K_2_HPO_4_, 1 g/L MgSO_4_·7H_2_O and 0.1 g/L MnSO_4_·H_2_O.

After selecting the most efficient strain, the effect of nutrient supplementation was assessed on that single strain by testing the addition of three nutrient solutions to the CWP. Nitrogen sources (ammonium chloride and yeast extract) and phosphorous (phosphate salts) are known to promote cell growth and ethanol production [33]. Magnesium has been identified as an active component, which prolongs exponential growth, resulting in increased yeast cell mass and it also reduces the decline in fermentative activity [34, 35]. Sodium thioglycolate acts as a reducing agent and neutralizes possible toxic effects [36, 37]. Therefore, several combinations of these nutrients were evaluated to check whether they were necessary for fermentation. Three preparations, namely A (3 g/L yeast extract, 2 g/L NH_4_Cl, 2 g/L KH_2_PO_4_, and 2 g/L K_2_HPO_4_), B (1 g/L MgSO_4_·7H_2_O) and C (200 mg/L sodium thioglycolate) were assessed. Nutrients were autoclaved within the CWP medium, except magnesium and manganese salts, which were added as a microfiltered concentrate after autoclaving. CWP samples supplemented with eight different combinations of the three preparations (ABC, AB, AC, BC, A, B, C or none) were fermented.

All fermentations were carried out in 100-mL Erlenmeyer flasks containing 1.25 mL of inoculum and 48.75 mL of fermentation medium. Flasks plugged with foam stoppers were incubated at 35°C and 150 rpm in an Infors HT Minitron orbital shaker during 48 h after adjusting the pH to 6.0. Experiments were performed in duplicate.

### *Saccharomyces* strains and lactose whey permeate hydrolysis optimization

After testing the incapacity of the four selected *S*. *cerevisiae* strains listed in section *Yeast strains and culture conditions* to naturally metabolize the lactose present in the CWP (data no included), lactose hydrolysis was performed using a commercial β-galactosidase (Ha-Lactase 2100, enzymatic activity of 2100 NLU/g, Chr. Hansen Holding A/S, Hoersholm, Denmark). In order to optimize the hydrolysis, a complete central design (CCD) and response surface methodology (RSM) experiments were developed using CWP with an initial lactose concentration of L_i_ = 120 g/L lactose. Three variables were assessed: enzyme dosage, pH and time. The response variables considered were the final concentrations of glucose (G_f_) and galactose (Gal_f_) released expressed in g/L, as well as the hydrolysis efficiency (η_(G+Gal)/L_), which was calculated as follows:

$$\eta_{(G+Gal)/L}=\frac{G_{f}+{Gal}_{f}}{L_{i}}\times 100$$ (1)

Some characteristics of these RSM experiments are provided in Table A in S1 Appendix. The estimated regression coefficients for hydrolysis efficiency (%) and the analysis of variance results are reported in Tables B and C in S1 Appendix, respectively. A surface model was fitted and the resulting polynomial equation was used to estimate the optimal enzyme dosage, time and pH values to obtain the highest amount of glucose and galactose released in the broth before the subsequent fermentation. Contour plots can be seen in Fig A in S1 Appendix for each pair of variables. A maximum lactose hydrolysis efficiency of 85% was obtained adding 0.28 mL/L of enzyme to the substrate and keeping the mixture at a pH of 5.9 and temperature of 30°C during 7 hours. The hydrolysate contained 65 g/L glucose, 65 g/L galactose and 7 g/L residual lactose.

In order to select the most efficient *S*. *cerevisiae* strain in the same conditions of *K*. *marxianus*, the four strains were compared for the fermentation of a hydrolyzed CWP without any nutrient supplementation. Fermentations were carried out in 100-mL Erlenmeyer flasks containing 50 mL of hydrolyzed medium and 0.1% (w/v) of inoculum. Flasks plugged with foam stoppers were incubated at 35°C and 150 rpm in an Infors HT Minitron orbital shaker (Infors AG, Bottmingen, Switzerland) during 65 h after adjusting the pH to 5.4. Experiments were performed in triplicate.

### Optimization of fermentation conditions

The optimization of operating conditions was made by employing each of the selected *K*. *marxianus* and *S*. *cerevisiae* strains, which had the best alcoholic performance in the strain comparison experiments.

Fermentations were carried out in 250-mL Erlenmeyer flasks containing 95 mL of CWP with an initial lactose concentration of 132.5 g/L (with previous lactose hydrolysis in the case of *S*. *cerevisiae*). The batch runs started after the aseptic addition of a ratio of 0.1% (w/v) of inoculum to the fermentation medium. Three variables were optimized: temperature (T,°C), pH (pH,—) and fermentation time (t, h). A complete central design (CCD) experiment was run and response surface methodology (RSM) was applied for evaluating the empirical model. Second order polynomials were fitted for each response and the resulting equations were used to estimate the optimal temperature, pH and time values that maximize the ethanol production (concentration and yield expressed per unit of lactose consumed). All the estimated optimal points were validated experimentally.

Twenty experiments were performed and included 8 cube points, 6 central points and 6 axial points (α = 1.68179). Fermentation conditions were simultaneously optimized for maximizing the responses of ethanol final concentration (E_f_), ethanol yield factor (Y_E/L_), ethanol profit factor (π_E_) and ethanol volumetric productivity (W_E_).

### Comparison of immobilization supports

**Fig 1:**
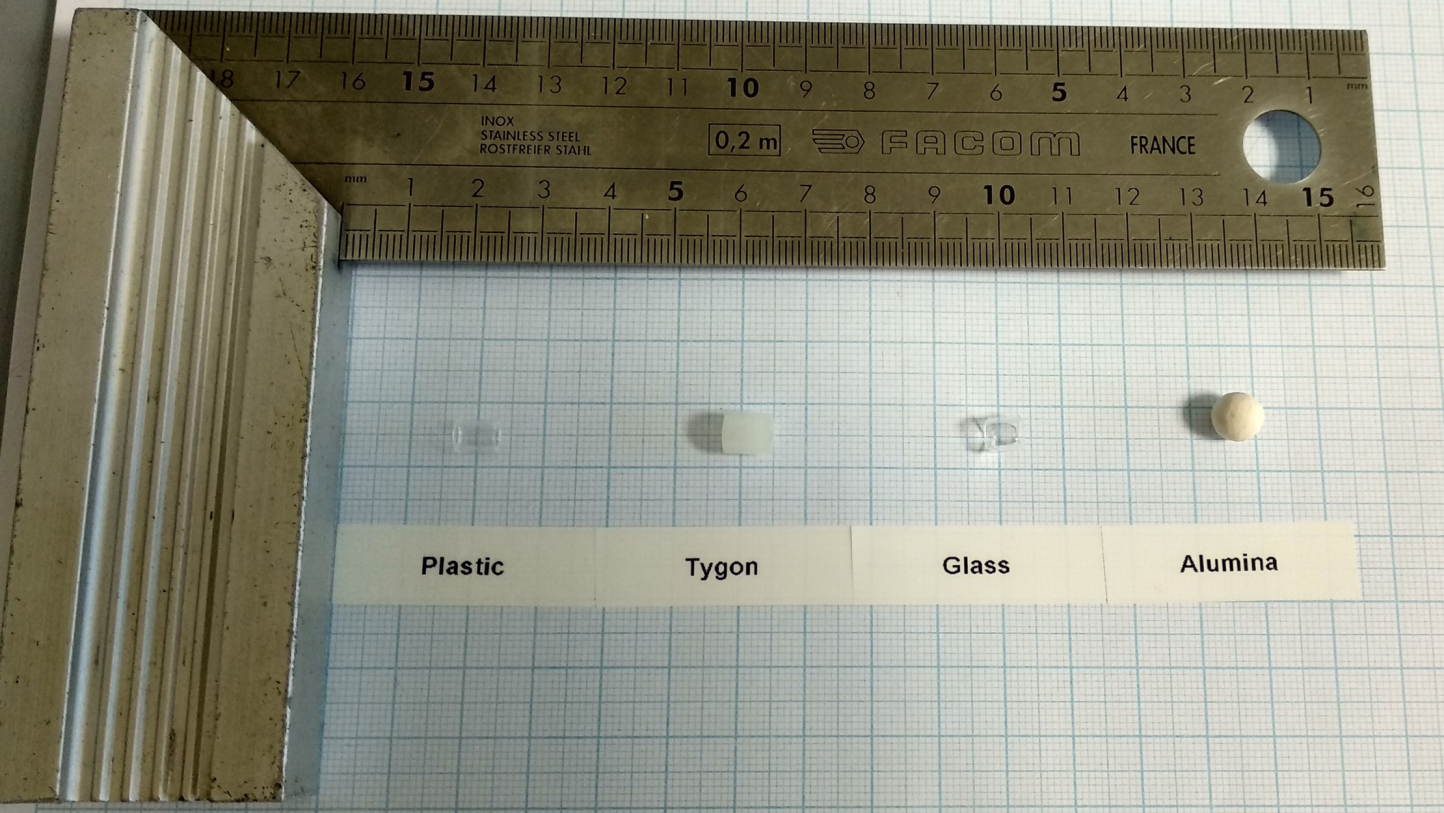
Inert supports used for cell immobilization. From left to right: plastic Raschig rings, Tygon silicone Raschig rings, glass Raschig rings and alumina beads.

Four inorganic porous materials were selected because of their wide availability, easy preparation and reutilization, their steam sterilizability and their inexpensive or low-cost. Raschig rings of similar dimensions (length of 5–5.5 mm) of three different porous materials (plastic, glass and Tygon silicone) and alumina beads with 5 mm of diameter were selected. A sample of the inert supports employed is shown in Fig 1. The inorganic porous supports were washed with deionized water and sterilized by autoclaving at 121°C for 15 minutes.

Approximately, 10 g of every support were introduced in 250-mL Erlenmeyer flasks, 50 mL of non-supplemented CWP with an initial lactose concentration of 130 g/L and 3.7% (v/v) of the selected yeast cells. The yeast strain and the fermentation conditions were obtained during the optimization step. For laboratory personal logistic needs, every 48 or 72 hours, fresh CWP replaced the exhausted whey medium for 7 cycles. Samples of exhausted whey were analyzed for fermentation performance. All experiments were performed in triplicate.

After 7 cycles, two supports were selected to test their stability for a prolonged period of time. The experiments were extended refreshing CWP during 14 cycles. In order to compare the effect of lactose load on immobilization performance, experiments were carried out employing non-supplemented CWP at two initial lactose concentrations (130 g/L and 170 g/L). All experiments were performed in triplicate.

### Chemical analyses of fermented broths

Periodic samples were collected from the fermentation flasks using aseptic techniques to measure lactose, glucose, galactose and ethanol concentrations. Samples were centrifuged at 12,000 × g in a micro-centrifuge for 3 min (MiniSpin, Eppendorf, Hamburg, Germany). The concentrations of lactose, ethanol and lactic acid production were measured from supernatant samples filtered through a 0.22 μm filter and analyzed by a high performance liquid chromatography (HPLC) system (Agilent LC1200 HPLC) coupled to a refractive index detector (Agilent 1200 Series), using a Bio-Rad Aminex HPX-87-H (300 mm x 7.8 mm) column (Bio-Rad, Hercules, California, USA) with 5 mM H_2_SO_4_ mobile phase, a flow rate of 0.6 mL/min and a column temperature of 60°C [21].

Fermentation kinetic parameters were calculated at the end of the runs as follows. Lactose consumption rate (ΔL) was defined as the rate of consumed lactose being L_i_ and L_f_, the initial and final concentration of lactose (g/L), respectively (Eq 2). The ethanol yield factor (Y_E/L,_ g/g) was defined as the ratio between ethanol final concentration (E_f_, g/L) and lactose consumed (g/L) (Eq 3). The yield conversion efficiency (η_E_, %) was defined as the ethanol yield versus the theoretical ethanol yield, assuming a theoretical ethanol production, by means of alcoholic fermentation, of 0.538 g of ethanol per g of lactose consumed by yeast [12] (Eq 4). Ethanol productivity (W_E_, g/ (L·h)) was defined as the ratio between ethanol concentration (g/L) and fermentation time (h) (Eq 5). In addition, we propose the use of a new parameter called profit factor (π_E_), defined as the ethanol concentration E_f_ (g/L) multiplied by the lactose consumption rate ΔL (Eq 6). The ethanol yield (Y_E/L_) and yield conversion efficiency (η_E_) can be misleading when low ethanol concentration (E_f_) and low lactose consumption (ΔL) values are recorded. From the environmental point of view, it is important to deplete as much lactose as possible during the fermentation, in order to reduce the COD of the broth before treating it as a liquid waste. Therefore, the parameter π_E_ combines in a single figure ethanol production and lactose consumption. In addition, high ΔL values imply a successful fermentation process with an almost complete use of available sugars by yeasts.

$$\Delta L=\left(\frac{L_{i}-L_{f}}{L_{i}}\right)$$ (2)

YEL=EfLi−Lf (3)

ηE=YEL0.538×100 (4)

$$W_{E}=\frac{E_{f}}{t}$$ (5)

$$\pi_{E}=E_{f}\times \Delta L$$ (6)

### Statistical analyses

Comparisons among treatments were assessed with a one-way ANOVA and the Tukey HSD test using the software Statistica 7 (StatSoft Inc., Tulsa, OK, USA); differences were considered significant when p < 0.05. For the optimization steps, experimental designs, such as Response Surface Methodology (RSM), were generated and interpreted with the software Minitab 16 (Minitab Inc., State College, PA, USA).

## Results and discussion

### *Kluyveromyces marxianus* strains and fermentation media comparison

**Fig 2:**
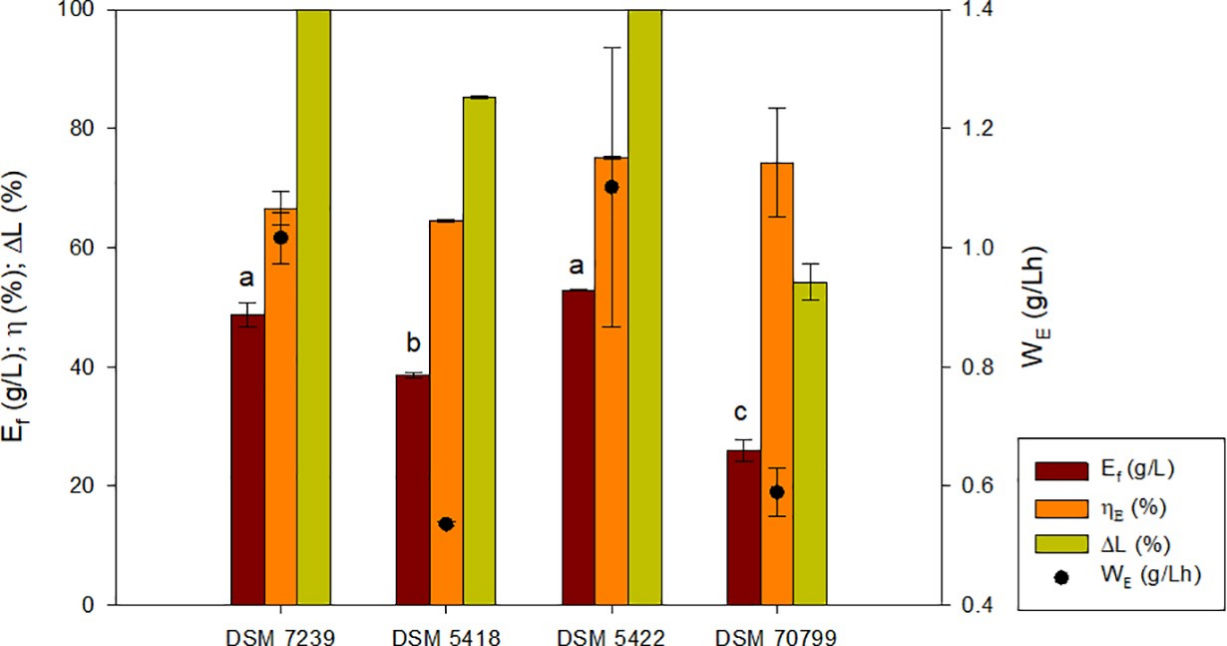
Fermentation performance of *K*. *marxianus* DSM 7239, DSM 5418, DSM 5422 and DSM 70799 in concentrated synthetic medium (L_i_ = 130 g/L). Parameters: final ethanol concentration (E_f_), ethanol yield conversion efficiency (η_E_), lactose consumption (ΔL) and productivity (W_E_). The different letters above ethanol bars (a, b, c) indicate the existence of significant differences (p < 0.05) among strains for ethanol production. If two strains share one letter, there are no significant differences between them.

This set of experiments was performed to determine the capacity of four different strains of *K*. *marxianus* to convert lactose into ethanol without inhibition’s problems working at lactose concentrations higher than 100 g/L. The four strains metabolized and biotransformed naturally lactose to ethanol. The ethanol produced, the lactose consumed and the ethanol yield varied substantially among the strains when a synthetic medium with an initial lactose concentration of 130 g/L was used. As can be observed in Fig 2, the highest ethanol production with a total lactose consumption corresponded to *K*. *marxianus* DSM 5422 and *K*. *marxianus* DSM 7239, with an ethanol yield of 0.41 g/g and 0.36 g/g, ethanol titers of 52.9 g/L and 48.8 g/L and productivities of 1.1 and 1.01 g/(L·h), respectively. Due to the better fermentation performance obtained with *K*. *marxianus* DSM 5422, this strain was selected for the subsequent optimization process to increase its ethanol production capacity.

**Fig 3:**
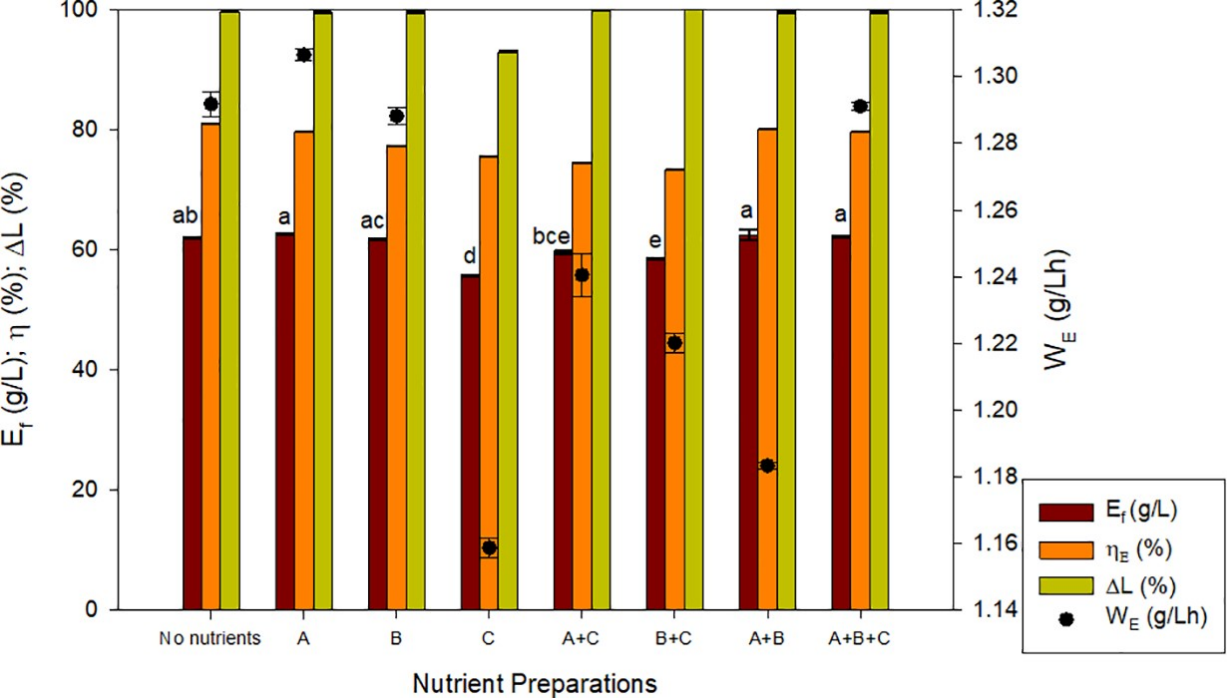
Evaluation of the effect of different combinations of nutrients on alcoholic fermentation performance. Response variables: Ethanol final concentration (E_f_), ethanol yield conversion efficiency (η_E_), lactose consumption (ΔL) and productivity (W_E_) of *K*. *marxianus* 5422 employing concentrated CWP (L_i_ = 130 g/L). Description of nutrient preparations: A (3 g/L yeast extract, 2 g/L NH_4_Cl, 2 g/L KH_2_PO_4_, and 2 g/L K_2_HPO_4_), B (1 g/L MgSO_4_·7H_2_O), C (200 mg/L sodium thioglycolate). The different letters above ethanol bars (a, b, c, d, e) indicate the existence of significant differences (p < 0.05) among samples for ethanol production. If two samples share one letter, there are no significant differences between them.

The effect of nutrient supplementation of CWP was tested with *K*. *marxianus* DSM 5422, due to its better performance employing synthetic media under the same fermentation conditions. Fig 3 shows the results of the fermentation of CWP without any supplementation and the eight combinations of nutrients tested. As it can be observed, the exclusive use of CWP without any supplementation provided a similar fermentation performance to that of CWP supplemented with solutions A, B, A+B and A+B+C. Due to the significant cost-saving, non-supplemented CWP was selected for the next experimental steps. The fermentation performance was: an ethanol titer of 62 g/L, an ethanol yield of 0.44 g/g (81.8% of the theoretical maximum yield) and a productivity of 1.3 g/(L·h). These results compare well with other works in literature using supplemented concentrated whey. Dragone *et al*. [20] reported conversions of concentrated deproteinized whey permeate (L_i_ = 150 g/L) into 55.9 g/L of ethanol with yields of 0.37 g/g employing *K*. *fragilis* Kf1. Kargi and Ozmihci [11] reported yields of 0.54 g/g with final ethanol concentrations of 81 g/L using *K*. *marxianus* NRRL-1195 strain in batch cultivations with a concentrated whey (150 g/L of lactose) and values of 3.7% (v/v) of ethanol with CWP (100 g/L of lactose) supplemented with 200 mg Na-thioglycolate to adjust the oxidation-reduction potential using *K*. *marxianus* DSM 7239 strains [11].

### *Saccharomyces cerevisiae* strains and fermentation media comparison

The capacity of four different strains of S. *cerevisiae* to produce ethanol from the hydrolyzate of high loaded lactose CWP (L_i_ = 140 g/L) was assessed. The ethanol produced, the sugars (glucose, galactose and residual lactose) consumed and the ethanol yield varied substantially among the strains. As can be observed in Table 1, the highest ethanol production and sugars consumption corresponded to Ethanol Red, the only strain that was able to metabolize galactose into ethanol. The ethanol titer was of 43.63 g/L with an ethanol yield of 0.345 g/g and a productivity of 0.70 g/(L·h).

**Table 1:** Fermentation performance of *S*. *cerevisiae* CECT 1383, Ethanol Red, CECT 13152 and Hércules in hydrolyzed CWP (L_i_ = 140 g/L).

Strain	E_f_ (g/L)	ΔL (%)	Δ(G+Gal) (%)	Y_E/L_ (g/g)	Y_E/(G+Gal)_ (g/g)	π_E_ (g/L)	W_E_ (g/L·h)	η_E_ (%)
CECT 1383 ^a^	25.11 ± 1.14	49.49 ± 0.31	51.19 ± 0.42	0.127 ± 0.007	0.129 ± 0.008	12.43 ± 0.52	0.39 ± 0.02	25.21 ± 1.48
Ethanol Red ^b^	45.63 ± 0.77	96.69 ± 0.03	99.52 ± 0.03	0.345 ± 0.006	0.350 ± 0.006	44.11 ± 0.73	0.70 ± 0.01	68.67 ± 1.14
CECT 13152 ^c^	34.24 ± 0.35	52.75 ± 1.11	55.29 ± 1.34	0.179 ± 0.001	0.180 ± 0.001	18.06 ± 0.53	0.53 ± 0.01	35.35 ± 0.21
Hércules ^c^	35.94 ± 0.96	54.30 ± 1.72	56.21 ± 1.87	0.192 ± 0.006	0.193 ± 0.007	19.53 ± 1.08	0.55 ± 0.01	37.91 ± 1.29

Parameters: final ethanol concentration (E_f_), sugar consumption (ΔL), ethanol yield factor (Y_E/S_), ethanol profit factor (π_E_), productivity (W_E_) and ethanol yield conversion efficiency (η_E_). The different superscript letters (a, b, c) indicate the existence of significant differences (p < 0.05) among strains for all the studied parameters. If two strains share one letter, there are no significant differences between them.

### Optimization of fermentation conditions

Optimal fermentation conditions (temperature; initial pH and time) were calculated via RSM experimental design for each strain employing CWP as feedstock.

#### *Kluyveromyces marxianus* strain

For the selected strain *K*. *marxianus* DSM 5422, the experimental conditions and the responses obtained are shown in Table 2. The full quadratic model was statistically effective for the final ethanol concentration at 95% confidence level. The estimated regression coefficients for ethanol titers (g/L) are reported in Table D in S1 Appendix. The analysis of variance results are shown in Table E in S1 Appendix. Fermentation conditions were simultaneously optimized for maximizing the most significant responses (E_f_ and π_E_) for the strain DSM 5422. According to the RSM mathematical estimations, a maximum ethanol titer of 58.2 g/L, an ethanol yield factor of 0.44 g/g with an ethanol profit factor (π_E_) of 58.2 g/L and an ethanol volumetric productivity (W_E_) of 1.33 g/(L·h) would be theoretically obtained at 30.3°C of temperature, initial pH 6.3 and 44 h of fermentation as optimal points. The theoretical conditions were tested experimentally to validate the model. An ethanol titer of 60.0 g/L was achieved for ethanol concentration with total lactose consumption; the ethanol yield factor Y_E/L_ was 0.45 g/g, corresponding with 85.3% of the theoretical yield. The ethanol profit factor was 60 g/L with an ethanol volumetric productivity of 1.23 g/(L·h). These data confirm the predictability of the fitted model for DSM 5422 strain, with a percentage error between experimental and predicted values of 3%.

**Table 2:** Experimental results of ethanol concentration (E_f_), ethanol yield factor (Y_E/L_), ethanol profit factor (π_E_) and ethanol volumetric productivity (W_E_) according to a central composite design employing *K*. *marxianus* DSM 5422 for the fermentation of CWP (L_i_ = 132.5 g/L).

	Variable Factor			Responses			
Run	pH	T	Time	E_f_	Y_E/L_	π_E_	W_E_
		(°C)	(h)	(g/L)	(g/g)	(g/L)	(g/(L·h))
1	6.3	37.5	36	54.19	0.41	54.10	1.51
2	7.5	45.0	24	33.05	0.24	21.69	1.38
3	6.3	37.5	36	53.1	0.40	52.76	1.50
4	6.3	37.5	15.8	45.44	0.40	36.70	2.87
5	5.0	30.0	48	56.43	0.46	56.43	1.18
6	6.3	37.5	56	47.28	0.36	47.19	0.84
7	5.0	30.0	24	44.94	0.48	34.49	1.87
8	6.3	37.5	36	51.70	0.39	49.08	1.51
9	5.0	45.0	48	20.86	0.45	7.97	0.43
10	7.5	30.0	48	50.94	0.43	50.94	1.06
11	5.0	45.0	24	23.98	0.44	10.65	1.00
12	6.3	50.1	36	10.78	0.43	2.05	0.30
13	7.5	45.0	48	26.76	0.40	15.16	0.56
14	7.5	30.0	24	39.64	0.44	29.95	1.65
15	6.3	37.5	36	51.10	0.39	50.34	1.50
16	6.3	37.5	36	54.77	0.41	54.01	1.52
17	8.3	37.5	36	51.08	0.41	41.56	1.42
18	4.1	37.5	36	44.40	0.45	32.93	1.23
19	6.3	37.5	36	50.24	0.39	49.70	1.40
20	6.3	24.9	36	52.92	0.42	50.02	1.47

Even though some authors have reported the effects of substrate and product inhibition on fermentation performance using *Kluyveromyves* strains with substrates at concentrations higher than 100 g/L lactose [38, 39], in this study *K*. *marxianus* DSM 5422 did not suffer substrate inhibition problems using CWP with an initial load of 132.5 g/L. Similar ethanol concentrations were reported using *K*. *marxianus* Kf1 at 30°C from 150 g/L of lactose under 44 h of fermentation [20]. However, ethanol concentration under optimal conditions is threefold higher than that reported by Ozmihci and Kargi [18] for *K*. *marxianus* DSM 7239, employing a lactose concentration of 130 g/L for an operation time of 48 h, working at 28°C and pH 5. This data agrees with reported values of ethanol yields of 0.53 g/g and 0.52 g/g, under hypoxic and anoxic conditions, respectively [40]. However, under aerobic conditions as those in the present study, values fell to 0.39 g/g [40], which are similar to the values reported by Dragone *et al*. [20].

The optimal operation time was 44 h, shorter than typically reported values ranging between 72 and 96 h when working with lactose loads lower than 100 g/L [10, 18, 41]. This work significantly contributes to improve the economy of this fermentation process. An increase of ethanol volumetric productivity between 30 and 60% was obtained in this work versus commonly reported productivity values of 0.5–0.9 g/(L·h) [18, 20, 38], using a non-supplemented high lactose load CWP (L_i_ = 132.5 g/L) as a substrate. The ethanol volumetric productivity of 1.23 g/(L·h) obtained in this work is similar to values reported by Saini *et al*. [42] employing the evolved adapted osmotolerant strain *K*. *marxianus* MTCC 1389.

On the other hand, the drawbacks of using directly *Kluyveromyces* strains to convert lactose to ethanol, including low ethanol titers of 2.5 to 4.2% (v/v), low osmotic tolerance and prolonged fermentation times [9, 43, 44], are overcome in this work. The strain *K*. *marxianus* DSM 5422 under the optimized conditions converted directly lactose to ethanol with ethanol titers of 6% (v/v) in shorter fermentation times (44 hours) employing high load lactose substrates with high osmotic tolerance. Thus, this study concludes that *K*. *marxianus* DSM 5422 is a promising strain for producing high yields of ethanol from non-supplemented high load lactose CWP. Although temperature, pH and operating time are factors that significantly affect the fermentation process, temperature showed the strongest effect on all responses.

#### *Saccharomyces cerevisiae* strain

In the same way as the fermentation with *K*. *marxianus* DSM 5422 was optimized, optimal fermentation conditions (temperature; initial pH and time) were calculated via RSM experimental design for *S*. *cerevisiae* Ethanol Red. In this case, the feedstock employed was the CWP hydrolyzed according to the conditions pointed out in section *Saccharomyces strains and lactose whey permeate hydrolysis optimization*. No nutrients were supplemented in order to compare the fermentation performance of both species employing exactly the same feedstock. The experimental conditions and the responses obtained for each condition are shown in Table 3. The full quadratic model was statistically effective for the final ethanol concentration at 95% confidence level. The estimated regression coefficients for ethanol titers (g/L) and the analysis of variance results are reported in Tables F and G in S1 Appendix, respectively. The optimized fermentation conditions that simultaneously maximized E_f_ and π_E_ responses were 30.5°C of temperature, initial pH 5.4 and 60 hours of fermentation. The estimated ethanol titer was 48.5 g/L with an ethanol yield factor of 0.37 g/g and an ethanol profit factor of 48.5 g/L, corresponding with a conversion efficiency of 70.1% and an ethanol volumetric productivity of 0.81 g/(L·h). To validate the fitted RSM model, the fermentation was experimentally tested at the optimized conditions. An ethanol titer of 47 g/L was achieved with a mean glucose consumption of 99.4% and a mean galactose consumption of 97.3%; the ethanol yield factor Y_E/L_ was 0.37 g/g, corresponding with 68.2% of the theoretical yield. The ethanol productivity was 0.73 g/(L·h). These data confirm the predictability of the fitted model for Ethanol Red strain, with a percentage error between experimental and predicted values of 3%. These results are in accordance with reported results of Zhang *et al*. [45], who recorded ethanol yield factors of 80% fermenting glucose synthetic media using *S*. *cerevisiae* BY4742 with a sugar load of 120 g/L after 72 hours of fermentation. These authors observed a strong effect of substrate concentration on ethanol yield, establishing the critical substrate concentration in 160 g/L of sugar. The high concentration of substrate decreased membrane fluidity and caused cell atrophy and organelle dehydration [45].

**Table 3:** Experimental results of ethanol concentration (E_f_), ethanol yield factor (Y_E/L_), ethanol profit factor (π_E_) and ethanol volumetric productivity (W_E_) according to a central composite design employing *S*. *cerevisiae* Ethanol Red for the fermentation of hydrolyzed CWP (L_i_ = 132.5 g/L).

Run	Variable Factor			Responses			
	pH	T	Time	E_f_	Y_E/L_	π_E_	W_E_
		(°C)	(h)	(g/L)	(g/g)	(g/L)	(g/(L·h))
1	5.5	40	72	22.52	0.27	7.563	0.31
2	5.0	35	64	45.03	0.35	44.72	0.70
3	4.5	30	72	44.70	0.35	44.46	0.62
4	5.8	35	64	47.75	0.37	47.46	0.75
5	5.5	30	56	45.65	0.38	40.10	0.82
6	5.0	43.4	64	19.25	0.30	0.70	0.30
7	5.5	40	56	35.61	0.33	23.87	0.64
8	5.0	35	64	45.36	0.35	45.04	0.71
9	5.5	30	72	47.17	0.36	46.93	0.66
10	4.5	40	72	17.67	0.25	1.83	0.25
11	5.0	35	77.5	42.50	0.33	42.24	0.54
12	5.0	35	50.6	39.24	0.36	27.96	0.78
13	4.2	40	64	28.09	0.29	14.44	0.44
14	4.5	40	56	28.31	0.30	13.64	0.51
15	5.0	35	64	44.77	0.35	42.31	0.70
16	5.0	35	64	46.49	0.36	46.13	0.73
17	5.0	35	64	45.20	0.36	43.32	0.71
18	5.0	26.6	64	43.92	0.35	41.35	0.69
19	4.5	30	56	41.79	0.36	33.49	0.75
20	5.0	35	64	45.90	0.36	43.99	0.72

Moreover, the enzymatic hydrolysis of lactose into glucose and galactose can cause catabolite repression [46], besides of being not economically convenient. Strains affected by such phenomenon show slower fermentations of sugar mixtures, such as glucose and galactose, compared to strains without catabolite repression [47].

Therefore, the use of *K*. *marxianus* DSM 5422 is clearly preferable over *S*. *cerevisiae* Ethanol Red for the fermentation of high lactose-loaded CWP.

### Comparison of immobilization supports

In order to determine the effect of the immobilization on inorganic supports on the bioconversion of *K*. *marxianus* DSM 5422 for long-term fed-batch processes using high loaded non-supplemented CWP as a substrate to produce ethanol, four inorganic supports (glass Raschig rings, plastic Raschig rings, Tygon silicone Raschig rings and alumina beads) were tested. The fermentation conditions were defined according to the results of the RSM model for this strain: 30.3°C for temperature, 6.3 for pH and 44 h for fermentation time.

**Fig 4:**
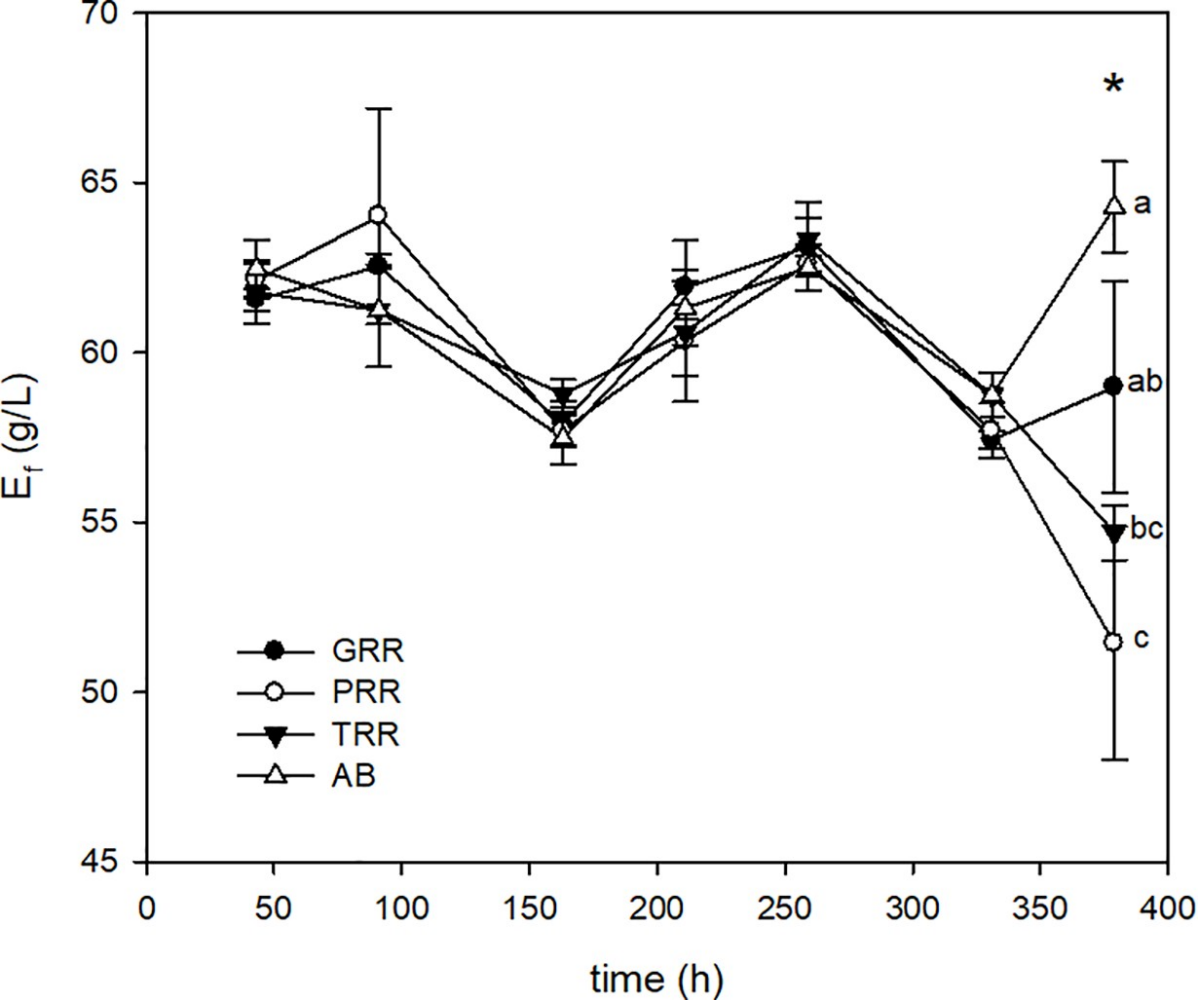
Evolution of ethanol concentration during ethanol fermentation for inorganic supports [glass Raschig rings (GRR), plastic Raschig rings (PRR), tygon Raschig rings (TRR) and alumina beads (AB)] during 7 fermentation cycles (L_i_ = 130 g/L) employing *K*. *marxianus* DSM 5422. Asterisks indicate the existence of significant differences (p < 0.05) among supports for a given batch. If two supports share one letter (a, b or c), there are no significant differences between them.

The profiles of ethanol production and the conversion efficiency for the four supports over time are shown in Fig 4. During the first cycles, the four inorganic supports had a similar behavior with final ethanol concentrations about 60 g/L. However, it was observed that the production of ethanol decreased drastically after the 6^th^ cycle with the supports of Tygon and plastic. On the contrary, the samples with glass Raschig rings and alumina beads, even though they suffered an ethanol production drop during cycle 6^th^, resumed production in the next batch. Conversion yields (η_E_) were higher than 80% per all cycles using inorganic supports, with yields higher than 90% for glass Raschig rings and alumina beads in the 7^th^ cycle.

**Fig 5:**
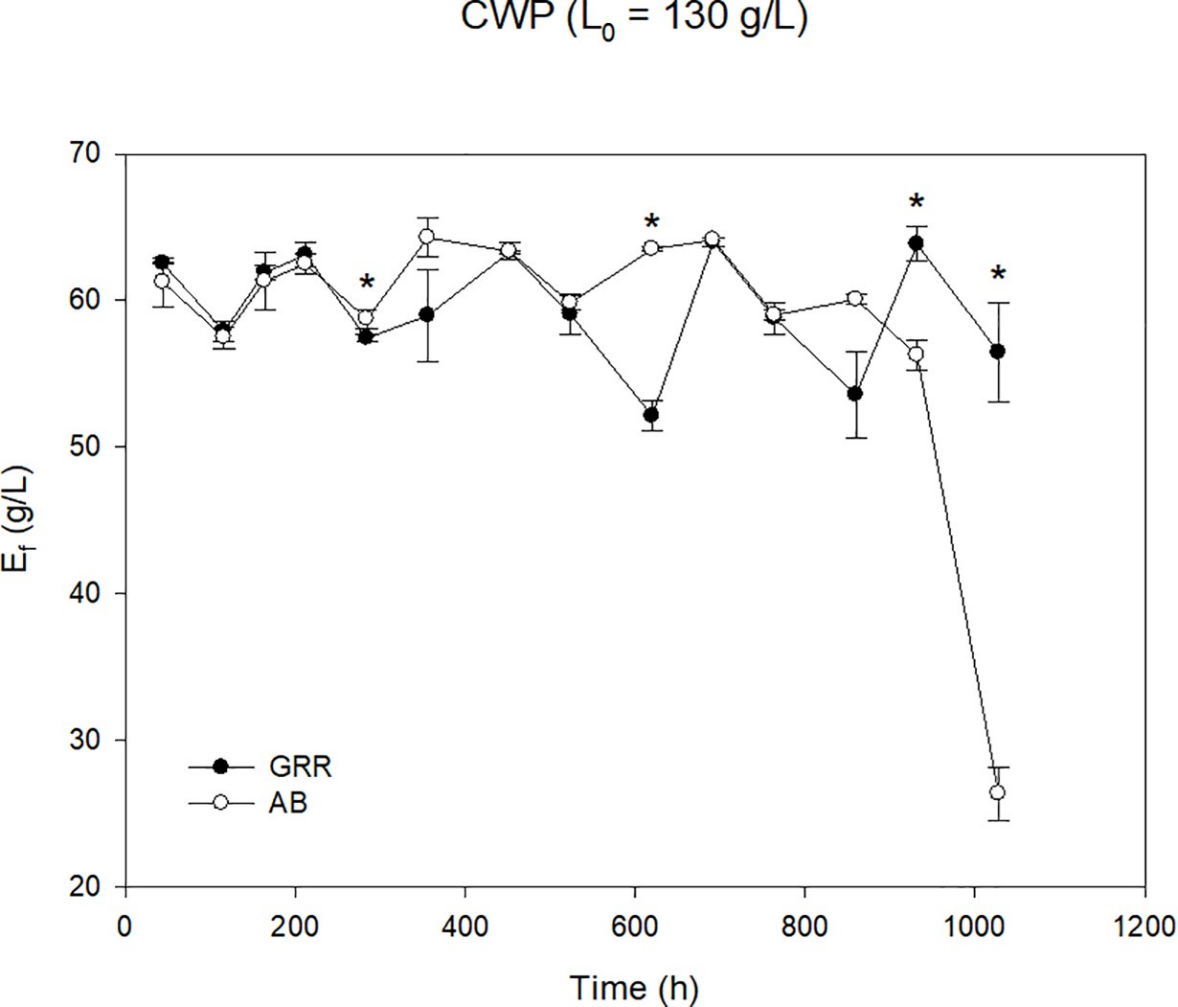
Evolution of ethanol concentration employing *K*. *marxianus* DSM 5422 on inorganic supports [glass Raschig rings (GRR) and alumina beads (AB)] during 14 fermentation cycles (L_i_ = 130 g/L). Asterisks indicate the existence of significant differences (p < 0.05) between supports for a given batch.

**Fig 6:**
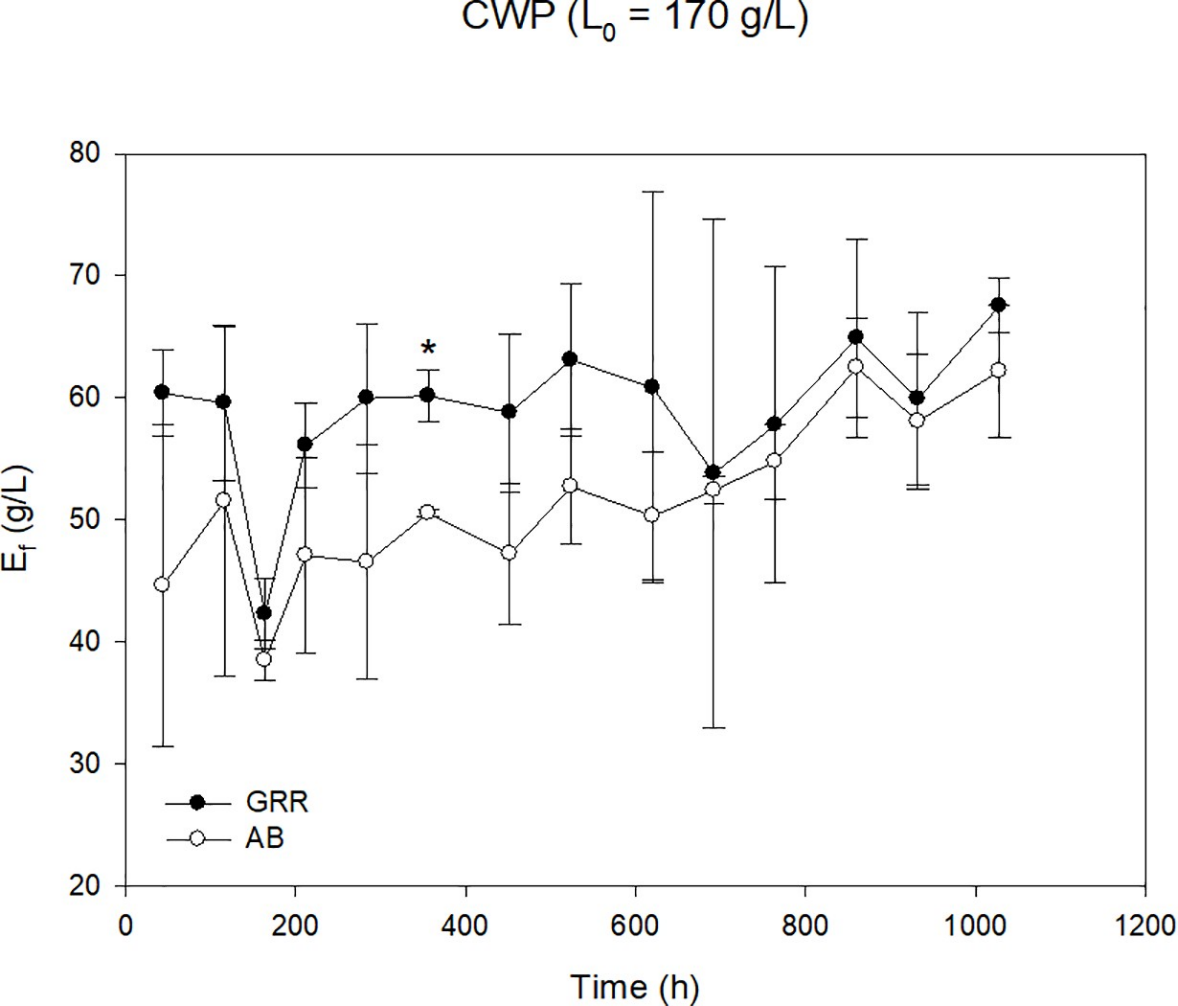
Evolution of ethanol concentration employing *K*. *marxianus* DSM 5422 on inorganic supports [glass Raschig rings (GRR) and alumina beads (AB)] during 14 fermentation cycles (L_i_ = 170 g/L). Asterisks indicate the existence of significant differences (p < 0.05) between supports for a given batch.

Due to their higher stability over time, glass Raschig rings and alumina beads were selected as immobilization supports to compare their behavior during a prolonged operation period of more than 1,000 hours, working in 14 cycles refreshing CWP with two different loads of lactose of 130 g/L and 170 g/L. For both inorganic supports, the profiles of ethanol production are presented in Fig 5 (L_i_ = 130 g/L) and Fig 6 (L_i_ = 170 g/L).

The experimental results showed that *K*. *marxianus* DSM 5422 was able to metabolize more than 90% of the lactose present in the broth to produce ethanol in 48–72 hours of fermentation during the 14 operational cycles, working with a CWP with 130 g/L lactose load (Fig 5). However, the strain only metabolized 80% of this disaccharide when the CWP was loaded at L_i_ = 170 g/L, independent of the support (Fig 6). For this reason, from the 7^th^ cycle, the fermentation time was increased to 72–96 hours for this high loaded CWP.

As it can be observed in Fig 5, in general the ethanol production remained constant at a value of 60 g/L during the 14 cycles for both supports, alumina beads and glass Raschig rings, working with a CWP with a lactose load of 130 g/L. The best performance was obtained with the alumina beads during all tested cycles with the exception of the 14^th^ cycle, when a significant ethanol concentration drop was observed.

In the case of CWP loaded with 170 g/L lactose (Fig 6), the alumina beads had a worse performance than the glass Raschig rings during all the experimental cycle but their behavior was improving over time. The mean ethanol production was of 58.2 g/L after 14 cycles prolonging during more than 1,000 hours of fermentation.

The mean fermentation kinetic parameters for each inorganic support at the two tested CWP loads (130 g/L and 170 g/L) during the 14 cycles are summarized in Table 4. An important decrease of all the kinetic parameters was observed working with lactose concentrations of 170 g/L, due to possible inhibitions by substrate. It is noteworthy that lactose consumption decreased in almost 25%, affecting significantly the profit factor (π_E_) and the productivity (W_E_). In base of the results, it would be more convenient to work with CWPs with lactose concentration lower than 170 g/L in order to get a total lactose depletion and avoid environmental problems.

**Table 4:** Comparison of mean fermentation parameters for *K*. *marxianus* DSM 5422 during the 14 cycles employing CWP with lactose loads of 130 g/L and 170 g/L for glass Raschig rings (GRR) and alumina beads (AB) supports.

		E_f_ (g/L)	Y_E/L_ (g/g)	η_E_ (%)	ΔL (%)	W_E_ (g/L·h)	π_E_ (g/L)
CWP (L_i_ = 130 g/L)	GRR	59.13	0.452	83.98	97.66	1.09	57.96
	AB	58.43	0.450	83.60	96.00	1.09	57.21
CWP (L_i_ = 170 g/L)	GRR	56.71	0.449	81.93	75.66	0.82	43.84
	AB	50.94	0.445	82.64	68.07	0.72	35.57

Ethanol final concentration (E_f_), ethanol yield factor (Y_E/L_), ethanol yield conversion efficiency (η_E_), lactose consumption (ΔL), ethanol profit factor (π_E_) and ethanol volumetric productivity (W_E_)

## Conclusions

High loaded cheese whey permeate (CWP) could be a perfect feedstock for ethanol fermentation, contributing simultaneously to an efficient reuse of the main waste stream of the dairy industry. The selection of an appropriate yeast strain is basic to overcome current techno-economical process difficulties including low ethanol titers, low osmotic tolerance and prolonged fermentation times. After the screening of eight yeast strains of the genera *Saccharomyces* and *Kluyveromyces*, the best performance was obtained employing *K*. *marxianus* DSM 5422, which was capable of fermenting directly high lactose-load CWP (> 130 g/L) to ethanol without the need of adding nutrients to the fermentation broth. The statistical optimization of fermentation conditions (temperature, initial pH and time) allowed the maximization of the fermentation performance (ethanol titer, ethanol yield and lactose consumption). Ethanol titers of 6% (v/v) and a total consumption of lactose in only 44 h were attained. Moreover, the feasibility of immobilizing this yeast strain on inorganic supports was assessed reporting stable ethanol production, yielding ethanol titers of 60 g/L and productivities of 1.09 g/(L·h) for 1,000 hours of operation (i.e. fourteen consecutive cycles), which remarkably reduces yeast cultivation costs. Economic and scale-up studies are needed to verify the feasibility of the proposed process.

## Supporting information

S1 Appendix (DOCX)
