# Supplementary material for: Yeast screening and cell immobilization on inert supports for ethanol production from cheese whey permeate with high lactose loads
Source: PLoS One. 2018 Dec 31;13(12):e0210002. doi: 10.1371/journal.pone.0210002 (PMC6312371; doi:10.1371/journal.pone.0210002)
Supplement: S1 Appendix — (DOCX) [file pone.0210002.s001.docx]

**Supplementary Material (SM)**

Table A. Experimental results of glucose (G_f_) and galactose (Gal_f_) release and hydrolysis efficiency (η_(G+Gal)/L_) according to a central composite design.

| Run | Variable Factor | | | *Responses* | | |  |
| --- | --- | --- | --- | --- | --- | --- | --- |
|  | pH  (-) | Lactase dosage | Time | G_f_ | Gal_f_ | η_(G+Gal)/L_ | |
|  |  | (μL) | (h) | (g/L) | (g/L) | (%) | |
| 1 | 6.00 | 100.00 | 4 | 28.94 | 22.55 | 42.44 | |
| 2 | 5.63 | 175.00 | 7 | 45.42 | 34.11 | 65.55 | |
| 3 | 5.63 | 175.00 | 7 | 45.44 | 34.16 | 65.61 | |
| 4 | 6.00 | 250.00 | 10 | 45.54 | 34.21 | 65.74 | |
| 5 | 5.63 | 175.00 | 7 | 45.42 | 34.11 | 65.55 | |
| 6 | 5.63 | 175.00 | 2 | 44.33 | 31.29 | 62.33 | |
| 7 | 5.63 | 175.00 | 7 | 44.49 | 33.56 | 64.33 | |
| 8 | 5.25 | 250.00 | 4 | 18.88 | 13.87 | 26.99 | |
| 9 | 5.63 | 48.87 | 7 | 22.18 | 19.89 | 34.68 | |
| 10 | 5.25 | 100.00 | 10 | 5.51 | 8.64 | 11.66 | |
| 11 | 6.26 | 175.00 | 7 | 45.15 | 33.97 | 65.22 | |
| 12 | 5.63 | 175.00 | 12 | 44.37 | 34.32 | 64.86 | |
| 13 | 5.63 | 175.00 | 7 | 44.22 | 33.17 | 63.79 | |
| 14 | 5.63 | 301.13 | 7 | 56.36 | 44.77 | 83.36 | |
| 15 | 4.99 | 175.00 | 7 | 2.48 | 4.46 | 5.72 | |
| 16 | 6.00 | 250.00 | 4 | 46.87 | 34.00 | 66.66 | |
| 17 | 5.25 | 250.00 | 10 | 18.26 | 15.09 | 27.49 | |
| 18 | 6.00 | 100.00 | 10 | 31.26 | 25.72 | 46.97 | |
| 19 | 5.63 | 175.00 | 7 | 35.53 | 26.49 | 51.12 | |
| 20 | 5.25 | 100.00 | 4 | 6.94 | 7.81 | 12.16 | |

Table B. Estimated regression coefficients of lactose hydrolysis efficiency (η_(G+Gal)/L_). Note: This model includes all the terms.

| Term | Coefficient | Standard error coef. | T | p | |
| --- | --- | --- | --- | --- | --- |
| Constant | 43.7453 | 2.686 | 16.285 | 0.000 | |
| Time (h) | -0.0727 | 1.782 | -0.041 | 0.968 | |
| Initial pH | 12.7981 | 1.782 | 7.181 | 0.000 | |
| Enzyme dosage (μL) | 8.3756 | 1.782 | 4.699 | 0.001 | |
| Time (h) * time (h) | -1.7973 | 1.735 | -1.036 | 0.325 | |
| Initial pH * Initial pH | -9.0575 | 1.735 | -5.221 | 0.000 | |
| Enzyme dosage (μL) * Enzyme dosage (μL) | -3.5933 | 1.735 | -2.071 | 0.065 | |
| Time (h) * initial pH | 0.3800 | 2.329 | 0.163 | 0.874 | |
| Time (h) * Enzyme dosage (μL) | -0.3550 | 2.329 | -0.152 | 0.882 | |
| Initial pH * Enzyme dosage (μL) | 0.9400 | 2.329 | 0.404 | 0.695 | |
|  |  |  |  |  | |
| S = 6.58633 | PRESS = 2814.42 |  |  |  | |
| R-square = 91.20 % | R-square(pred.) = 42.94 % | R-square(adj.) = 83.29 % | | |  |

Table C. Analysis of variance of lactose hydrolysis efficiency (η_(G+Gal)/L_). Note: This model includes all the terms.

| Source | df | Sum of squares Seq. | Sum of squares Adjust. | Mean squares Adjust. | F | p |
| --- | --- | --- | --- | --- | --- | --- |
| Regression | 9 | 4498.41 | 4498.41 | 499.82 | 11.52 | 0.000 |
| Lineal | 3 | 3194.98 | 3194.98 | 1064.99 | 24.55 | 0.000 |
| Time (h) | 1 | 0.07 | 0.07 | 0.07 | 0.00 | 0.968 |
| Initial pH | 1 | 2236.88 | 2236.88 | 2236.88 | 51.57 | 0.000 |
| Enzyme dosage (μL) | 1 | 958.03 | 958.03 | 958.03 | 22.08 | 0.001 |
| Quadratic | 3 | 1294.20 | 1294.20 | 431.40 | 9.94 | 0.002 |
| Time (h) * time (h) | 1 | 6.29 | 46.55 | 46.55 | 1.07 | 0.325 |
| Initial pH * Initial pH | 1 | 1101.83 | 1182.28 | 1182.28 | 27.25 | 0.000 |
| Enzyme dosage (μL) *  Enzyme dosage (μL) | 1 | 186.08 | 186.08 | 186.08 | 4.29 | 0.065 |
| Interaction | 3 | 9.23 | 9.23 | 3.08 | 0.07 | 0.974 |
| Time (h) * initial pH | 1 | 1.16 | 1.16 | 1.16 | 0.03 | 0.874 |
| Time (h) * Enzyme dosage  (μL) | 1 | 1.01 | 1.01 | 1.01 | 0.02 | 0.882 |
| Initial pH * Enzyme dosage  (μL) | 1 | 7.07 | 7.07 | 7.07 | 0.16 | 0.695 |
| Residual error | 10 | 433.80 | 433.80 | 43.38 |  |  |
| Lack of fit | 5 | 357.68 | 357.68 | 71.54 | 4.70 | 0.057 |
| Pure error | 5 | 76.12 | 76.12 | 15.22 |  |  |
| Total | 19 | 4932.21 |  |  |  |  |

Table D. Estimated regression coefficients of ethanol (g/L) for strain *K. marxianus* DSM 5422. Note: This model includes all the terms.

| Term | Coefficient | Standard error coef. | T | p | |
| --- | --- | --- | --- | --- | --- |
| Constant | 52.6545 | 1.2594 | 41.8110 | 0.000 | |
| Temperature (°C) | -11.5818 | 0.8356 | -13.8610 | 0.000 | |
| Initial pH | 1.1287 | 0.8356 | 1.3510 | 0.207 | |
| Time (h) | 1.2063 | 0.8356 | 1.4440 | 0.179 | |
| Temperature (°C) * Temperature (°C) | -8.2077 | 0.8134 | -10.0910 | 0.000 | |
| Initial pH * Initial pH | -2.5897 | 0.8134 | -3.1840 | 0.010 | |
| time (h) * time (h) | -3.0777 | 0.8134 | -3.7840 | 0.004 | |
| Temperature (°C) * Initial pH | 3.2200 | 1.0917 | 2.9500 | 0.015 | |
| Temperature (°C) * time (h) | -4.0250 | 1.0917 | -3.6840 | 0.004 | |
| Initial pH * time (h) | -0.4200 | 1.0917 | -0.3850 | 0.708 | |
|  |  |  |  |  | |
| S = 3.08780 | PRESS = 624.161 |  |  |  | |
| R-square = 97.08 % | R-square(pred.) = 80.85 % | R-square(adjusted) = 94.44 % | | |  |

Table E. Analysis of variance of Ethanol (g/L) for strain *K. marxianus* DSM 5422. Note: This model includes all the terms.

| Source | df | Sum of squares Seq. | Sum of squares Adjust. | Mean squares Adjust. | F | p |
| --- | --- | --- | --- | --- | --- | --- |
| Regression | 9 | 3164.68 | 3164.68 | 351.63 | 36.88 | 0.000 |
| Lineal | 3 | 1869.17 | 1869.17 | 623.03 | 65.35 | 0.000 |
| Temperature (°C) | 1 | 1831.9 | 1831.9 | 1831.9 | 192.13 | 0.000 |
| Initial pH | 1 | 17.4 | 17.4 | 17.4 | 1.82 | 0.207 |
| Time (h) | 1 | 19.87 | 19.87 | 19.87 | 2.08 | 0.179 |
| Quadratic | 3 | 213.96 | 213.96 | 71.32 | 7.48 | 0.006 |
| Temperature (°C) * Temperature (°C) | 1 | 908.85 | 970.84 | 970.84 | 101.82 | 0.000 |
| Initial pH * Initial pH | 1 | 36.19 | 96.65 | 96.65 | 10.14 | 0.010 |
| time (h) * time (h) | 1 | 136.5 | 136.5 | 136.5 | 14.32 | 0.004 |
| Interaction | 3 | 213.96 | 213.96 | 71.32 | 7.48 | 0.006 |
| Temperature (°C) * Initial pH | 1 | 82.95 | 82.95 | 82.95 | 8.7 | 0.002 |
| Temperature (°C) * time (h) | 1 | 129.61 | 129.61 | 129.61 | 13.59 | 0.004 |
| Initial pH * time (h) | 1 | 1.41 | 1.41 | 1.41 | 0.15 | 0.708 |
| Residual error | 10 | 95.35 | 95.35 | 9.53 |  |  |
| Lack of fit | 5 | 79.27 | 79.27 | 15.85 | 4.93 | 0.052 |
| Pure error | 5 | 16.07 | 16.07 | 3.21 |  |  |
| Total | 19 | 3260.05 |  |  |  |  |

| Term | Coefficient | Standard error coef. | T | p |
| --- | --- | --- | --- | --- |
| Constant | 45.0389 | 1.3947 | 32.2930 | 0.000 |
| Temperature (°C) | -8.6176 | 0.9253 | -9.3130 | 0.000 |
| Initial pH | 3.8474 | 0.9253 | 4.1580 | 0.002 |
| Time (h) | -1.0850 | 0.9253 | 4.1580 | 0.002 |
| Temperature (°C) * Temperature (°C) | -4.9045 | 0.9008 | -5.4450 | 0.000 |
| Initial pH * Initial pH | -2.6647 | 0.9008 | -2.9580 | 0.140 |
| Time (h) * Time (h) | -1.6217 | 0.9008 | -1.8000 | 0.102 |
| Temperature (°C) * Initial pH | 0.6025 | 1.2090 | 0.4980 | 0.629 |
| Temperature (°C) * time (h) | -3.3950 | 1.2090 | -2.8080 | 0.019 |
| Initial pH * time (h) | -0.6050 | 1.2090 | -0.5000 | 0.628 |
|  |  |  |  |  |
| S = 3.41960 | PRESS = 765.074 |  |  |  |
| R-square = 93.77 % | R-square(pred.) = 59.22 % | R-square(adj.) = 88.16% | |  |

Table F. Estimated regression coefficients of Ethanol (g/L) for strain *S. cerevisiae* Ethanol Red. Note: This model includes all the terms.

Table G. Analysis of variance of Ethanol (g/L) for strain *S. cerevisiae* Ethanol Red. Note: This model includes all the terms.

| Source | df | Sum of squares Seq. | Sum of squares Adjust. | Mean squares Adjust. | F | p |
| --- | --- | --- | --- | --- | --- | --- |
| Regression | 9 | 1759.07 | 1759.07 | 195.45 | 16.71 | 0.000 |
| Lineal | 3 | 1232.45 | 1232.45 | 410.82 | 35.13 | 0.000 |
| Temperature (°C) | 1 | 1014.21 | 1014.21 | 1014.21 | 86.73 | 0.000 |
| Initial pH | 1 | 202.16 | 202.16 | 202.16 | 17.29 | 0.002 |
| Time (h) | 1 | 16.08 | 16.08 | 16.08 | 1.37 | 0.268 |
| Quadratic | 3 | 428.58 | 428.58 | 142.86 | 12.22 | 0.001 |
| Temperature (°C) *  Temperature (°C) | 1 | 299.45 | 346.64 | 346.64 | 29.64 | 0.000 |
| Initial pH * Initial pH | 1 | 91.23 | 102.33 | 102.33 | 8.75 | 0.014 |
| Time (h) * time (h) | 1 | 37.9 | 37.9 | 37.9 | 3.24 | 0.102 |
| Interaction | 3 | 98.04 | 98.04 | 32.68 | 2.79 | 0.095 |
| Temperature (°C) * Initial pH | 1 | 2.90 | 2.90 | 2.9 | 0.25 | 0.629 |
| Temperature (°C) * time (h) | 1 | 92.21 | 92.21 | 92.21 | 7.89 | 0.019 |
| Initial pH * time (h) | 1 | 2.93 | 2.93 | 2.93 | 0.25 | 0.628 |
| Residual error | 10 | 116.94 | 116.94 | 11.69 |  |  |
| Lack of fit | 5 | 96.93 | 96.93 | 19.39 | 4.85 | 0.054 |
| Pure error | 5 | 20.00 | 20.00 | 4.00 |  |  |
| Total | 19 | 1876 |  |  |  |  |

Fixed Values

Fixed Values

Fixed Values

Fig A. Contour plots for the estimation of lactose hydrolysis efficiency (η_(G+Gal)/L_) as a function of hydrolysis conditions (enzyme dosage, initial pH and time), according to the mathematical RSM model.
